# Examining oxyhydrogen gas generation experimentally using wet cell design

**DOI:** 10.1371/journal.pone.0324921

**Published:** 2025-06-03

**Authors:** M. S. Gad, A. K. El Soly, Subhav Singh, Kamal Sharma, Saurav Dixit, Md irfanul Haque Siddiqui

**Affiliations:** 1 Mechanical Engineering Department, Faculty of Engineering, Fayoum university, Fayoum, Egypt; 2 Mechanical Engineering Department, Faculty of Engineering, Al Azhar university, Cairo, Egypt; 3 Chitkara Centre for Research and Development, Chitkara University, Rajpura, Himachal Pradesh India; 4 Division of research and development, Lovely Professional University, Phagwara, Punjab, India; 5 Department of Mechanical Engineering, GLA university, Mathura, India; 6 Department of Mechanical Engineering, College of Engineering, King Saud University, Riyadh, Saudi Arabia; National Chung Cheng University, Taiwan & Australian Center for Sustainable Development Research and Innovation (ACSDRI), AUSTRALIA

## Abstract

Oxyhydrogen (HHO) gas, which is created when water is electrolyzed using a dry cell electrolyzer, is becoming more and more popular as a new energy source because of its improved combustion properties. The creation of HHO wet cell is the primary goal of the current study in order to maximize gas flow rate and improve electrolyzer efficiency compared to dry cell. An inexpensive electrolyzer made of local obtainable parts was used to create HHO. Stainless steel 316L electrodes having a surface area of 136.5 cm^2^ and 4 mm distance between plates were used to generate HHO gas. Various concentrations of KOH and NaOH electrolytes were used. The rate of HHO generation was influenced by the electrolyte ratio, operation time, cell connection, electric current, electrolyte temperature, and voltage. Different plate arrangements were looked at. It had been discovered that raising the voltage, electrolyte temperature, electrolyte concentration, and applied current all contributed to higher gas generation. At 90 min. operation time, the wet cell showed continuous output peak values of 975, 1160, 1325, and 1375 ml/min at 5, 10, 15, and 20 g/L NaOH, respectively, along with supply currents of 17.8, 23.5, 26, and 27 A. At 5, 10, 15, and 20 g/L catalyst percentage, the temperatures were increased to 35, 44, 50, and 58°C, respectively. With a production efficiency of 69.3%, the HHO wet cell generated 1160 ml per minute at 18 amps and 10 g/litre of NaOH. Wet cell electrolyzers overheating has drawback for practical applications due to higher current supply about dry cell.

## 1. Introduction

A renewable sustainable fuel is now necessary due to the depletion of fossil fuels and the sharp rise in harmful emissions. Global warming and climate change are caused by carbon dioxide emissions. Engines and heating systems can use hydroxy gas (HHO) as an alternative fuel [1]. A non-spontaneous redox reaction is fuelled by electrical energy during the electrolysis process. Water is electrochemically converted to hydrogen and oxygen by an ionization reaction that yields HHO. HHO has extremely high diffusivity due to the presence of hydrogen. Because HHO is less dense than other fuels and needs a larger storage capacity, hazardous situations should be prevented or minimized. There is a considerable chance of backfire due to the short quenching distance; however, this risk can be decreased by employing a flame arrestor. Oxyhydrogen gas is lighter than air and has no color or smell. The flame of HHO burns more quickly because it contains hydrogen. Electrolytes are added to water to boost its electrical conductivity. As electrode surface activity and efficiency increase, the electrical resistances and splitting reaction potential of the electrolyte drop [2]. Wet cells and dry cells are the two cell types that produce HHO. Alpha, beta, and omega cells are the three types of dry cell HHO producers. Iron, copper, graphite, stainless steel, and aluminum plates are frequently used to make electrodes. Electrolyte solutes are composed of sodium hydroxide (NaOH), potassium hydroxide (KOH), sodium bicarbonate (NaHCO_3_), sodium acetate (CH_3_COOH), and sodium chloride (NaCl). However, the anode and cathode on the cell plates are constructed from stainless steel grades 302, 304, and 316L. With a melting point between 400 and 1370 and a thermal conductivity of 16.3 W/m K, grade 316L is the ideal metal for electrolysis. Due to their high activity, electrical conductivity, and corrosion resistance, cobalt, nickel, aluminum, and Raney nickel are the most often used materials for electrodes [2].

Both acidic and alkaline electrolytic solutions are possible, while alkaline solutions are more frequently utilized since they reduce the risk of corrosion. Alkaline electrolysis can be achieved with either a monopolar or a bipolar electrode arrangement. The bipolar arrangement is more compact and utilizes less material. An electrolyte liquid contained in a water vessel was utilized to immerse the electrodes of wet cells. Wet cells are easier to construct, more stable, and produce gas more effectively. The positive electrodes in a wet cell experience increased corrosion, heat, and current. Water steam is produced with additional heat and used to restore the volume of hydrogen gas. Common rubber O-rings are used to keep the stainless steel 316L plates apart. The plates and sealing agent are covered with acrylic plastic. With overflow, a water trap and a gas dryer, HHO bubblers function as a secure automated water level [3]. The supply current is regulated by pulse width modulation, or PWM. It is advised to use 0.25 litres of HHO per minute for every litre of engine size. The three active plates of an alpha cell consist of one positive pole and two negative poles connected by external electrical connections. The working current is far less than expected since water has a high electrical resistance. The three active plates of an alpha cell consist of one positive pole and two negative poles connected by external electrical connections. Higher supply current is used by alpha cells to produce more HHO gas. Three active plates, fourteen 2 mm thick O-rings, and two 8 mm thick acrylic plastic pieces make up a beta cell. Neutral plates made of stainless steel 316L are used in omega cells. It is made up of five active plates, 26 2 mm thick O-Rings, and two 8 mm thick acrylic plastic pieces. By needing more time and space, the supplied current is reduced. It is believed that the best cell type for a car engine is the beta cell [3].

The efficiency of the cell grew as its temperature rose. The properties of the electrode material and the operating temperature have a major effect on the exchange current densities. As the current density increases, so does the Faraday efficiency. The amount of HHO produced increases over time in steady state. As electrolyte concentration rises, so does HHO generation [4]. Alkaline electrolysers, solid oxide, and polymer electrolyte membranes are the three types that are utilized. Because of their low working temperature and low production cost, alkaline electrolysers have been used for hydroxy formation [5]. Water can be converted into HHO more quickly by adding sodium bicarbonate gradually since the electrolysis process produces heat. The electrolyte facilitates the flow of electricity from the anode to the cathode [6]. Electrolysers have advantages and disadvantages, just like batteries. Ions can move in two different states: an aqueous solution (after dissolving in water) and a liquid state (after melting). Voltage is applied to the two electrodes, charging each neutral plate with a distinct polarity. The bubbler prevented the electrolyser from blowing up due to the backfire. A little amount of water containing moderately acidic vinegar was supplied to the electrolyser in order to neutralize any waste sodium hydroxide vapor in the generated gas. Backfire of bubbler water into the electrolyser was prevented by a check valve between the bubbler and electrolyser. Precautions, including using a flash back arrestor, which reverses the direction of the gas supply, should be taken to protect the system against damage and explosions. Because direct current has alower electrical impedance than alternating current, it is recommended to use it instead. The electrodes were made of carbon and magnesium. Maintaining a minimum separation between the electrodes is necessary to prevent void fracturing, even when electrical resistance decreases with decreasing distance [7].

Electrical resistance rises and efficiency falls as a result of gas bubbles. The electrical resistance decreases as the electrodes’ surface area increases. To let the bubble out, the electrodes are positioned vertically. Platinum plates perform better than molybdenum plates in KOH solution. The cost of HHO storage tanks is high. The strong diffusivity, low ignition energy, broad flammability limits, and short quenching distance of hydrogen flames increase the risk of explosion and flashback. Electrolyzers effectively create 40–70% HHO. Production efficiency can be increased by raising the temperature, current density, operating pressure, and electrolyte conductivity [8]. Since grades 302 and 304 stainless steel are advised for the cathode rather than the anode, grade 316L stainless steel ought to be utilized for the anode. When the electrolyte solution was heated up from the over current, more vapor bubbles were formed, burst and pitting the plates. The lower HHO generation rate was caused by smaller anode and cathode plate spacing [9]. Electrolyte replenishment is made possible by tiny holes and staggered cell design, which lessen efficiency loss from current leakage between cells. Compared to dry cells, wet cells are easier to manufacture, produce more gas, require larger dimensions, and have more anode electrode corrosion; nevertheless, they also consume more current and generate more heat. The extra heat generated by the overcurrent causes steam to replace the volume of HHO gas. Dry cells are preferred over wet cells due to their smaller size, simpler structure, and simpler manufacturing procedure. In contrast, the wet cell is less expensive than the dry cell. Wet cells produce more hydroxy gas than dry cells under the same design conditions [10]. Compared to distilled water, tap water is more prone to corrode. Wet electrodes corrode more than dry ones because a greater area of the plate is exposed to the electrolyte. While 0.75 LPM of HHO gas was utilized in the wet cell design, 0.5 LPM was continuously fed into the engine air intake manifold for the dry cell design. A petrol engine should run at the lowest possible hydroxy flow rate to prevent knocking and explosion. Applying the conventional HHO enrichment level, this was set at high rate of 1 LPM and cylinder capacity of 20% [11].

Each stack of connected plates contains an anode, a cathode, and a neutral. If there is a discernible increase in the electrolyte ratio as a result of heat generation, the current increases [12]. Bubble formation on the electrode surface increases the risk of overvoltage and internal resistance; mesh electrodes reduce this risk. High electrolyte content results in the greater conductivity and decreased electric potential [13,14]. Without putting the electrolyte at risk, nickel-plated electrodes are utilized to increase corrosion resistance and decrease cation production [15]. It has been demonstrated that potassium hydroxide (KOH) increases ion conductivity [16,17]. The highest amount of HHO is produced by KOH, which has a 20% concentration [18]. The molality of the catalyst increased to about 1% of mass, which resulted in an extreme drop in overall electrical resistance and a dramatic surge in battery current [19]. The current increases if the electrolyte content noticeably rises [20]. 10 LPM of oxyhydrogen gas was produced using a 30 Amp supply current. Pure hydrogen and HHO were mixed at a constant rate of 10 L/min [21]. Since H2 and HHO are interchangeable, HHO is used instead of H_2_ [22, 23, 24]. Demineralized or distilled water is used to make the electrolyte [25,26]. When distilled water and sodium hydroxide catalyst are used to produce an electrolyte of 10–15% m/m, the highest HHO flow rate could be achieved [27,28]. A fuzzy self-tuning PID controller is used to enhance hydroxy production while shielding the hydroxy generator from the effects of high temperatures [29, 30, 31]. The bubbles on the electrodes’ active surfaces are lessened by the application of magnetic fields, pulsed power, and ultrasonic waves. Pulsed DC electricity was used to increase the HHO cell’s efficiency [32]. The simulated HHO gas was made by combining oxygen and compressed hydrogen. To provide a constant supply pressure, these cylinders are equipped with pressure regulators [33]. After being powered by solar panels, a DC converter uses 20% KOH to transfer power to a 24 V, 60 Ah battery [34]. In the process of separating hydrogen and oxygen, KOH is preferred over NaOH due to its greater affinity for and solubility in water [35]. When the generator’s internal resistance is decreased, less power is required to overcome it, increasing the efficiency of gas generation [36]. The voltage is reduced because the detached electrodes act as bipolar devices and simultaneously have both polarities [37]. The rate at which HHO gas is produced depends on the kind of electrode material, electrode geometry, electrode gap, electrolyte type, electrolyte concentration, and passing current [38]. The amount of HHO gas produced depends on the current flowing over the plate surface area. The current was increased to increase the generation of gas [39,40]. In single-atom molecules, no interatomic connections should be broken. The shell is built of fuel, whereas the centre is made of water because to the differences in densities [41]. In order to create HHO, neutrals increase surface area and lower plate voltage [42]. NaOH has less efficiency than KOH [43]. The water tank should be about 70% filled. Destructive corrosion can occur when water vapour contains minute electrolyte particles. The only expenses related to the electrolysis process are the generator and accessories, which should cost about $200 depending on the equipment’s availability [44].

The issue of a limited oxygen supply can be resolved by using oxyhydrogen (HHO). It is completely safe to use HHO with on-board apps. Compared to pure hydrogen, HHO has better combustion characteristics because it incorporates oxygen. The ratio of hydrogen to oxygen by volume is 2:1. HHO may speed up combustion and enhance overall engine performance. Smaller cars’ economy can be increased by using HHO in petrol engines [45]. Onboard hydrogen storage is a major problem, though. As a result, on-demand HHO generation is recommended. The operational efficiency of HHO manufacturing systems ranges from 40% to 70%. Efficiency can be raised by increasing electric current, operating pressure, electrolyte solution conductivity, and electrode conditioning [46]. Because of its wide range of flammability, HHO can be used. HHO gas’s remarkable diffusivity promotes consistent fuel-air amalgamation and quickly diffuses leaks to prevent or reduce hazards. Oxyhydrogen can be produced using a variety of techniques, including electrolysis, thermolysis, and steam methane reforming. Electrolysis is the most widely used of these due to its ease of use, low energy consumption, reduced cost, reliability, durability, safety, and the possibility of producing 99.98% pure hydrogen. Fuel and energy efficiency are impacted by low HHO density, which increases the demand for storage. The considerable risk of backfire brought on by the short quenching distance can be reduced using a flame arrester. HHO must be used more safely because of its hydrogen component, which speeds up flames [47,48]. Hydrogen and HHO are produced via water electrolysis, which requires a high power supply. Renewable energy systems (RESs) including photovoltaic (PV) and wind turbines (WT) have recently been used to manufacture hydrogen [49].

The influence of generated hydroxy by dry cell using water electrolysis was the main focus of the literature review. There is a gap not covered by literature to produce oxyhydrogen gas using wet cell design. The wet design would boost HHO gas output effectively. Wet cells with the same design of dry type produce more HHO gas. The wet cell electrolyzer employs special design and electrode configuration of stainless material. The experiments demonstrated higher gas yield using the effects of these parameters as voltage, electrolyte concentration, plate spacing, and temperature. As a result, little is known about how various parameters impact the gas produced by wet cell. The main goal of this work is to build a wet cell that can produce the necessary quantity of HHO gas to replace engine fuel and to test the wet cell over an extended period of time to track changes in HHO gas evolution. HHO was generated via low-cost electrolyzer made of readily available and fairly priced components. The research’s goal is to generate the highest gas flow rate compared to dry type. Examining the consequences of the oxyhydrogen produced by wet cell water electrolysis was the primary goal of the research work. Consequently, further study is needed to show how various factors impact the gas production for wet cells, which remains unfilled. Effects of varying the concentration, electrode spacing, temperature, electrolyte type, gasket thickness, applied voltage, applied current, and operating time were conducted. The adjustments were done to dry cell to enhance performance and outlet yield. Prioritizing price or appropriateness for on-vehicle or small-scale HHO systems may help the study’s practical, sustainable energy applications. The design also incorporates easily accessible materials and investigates the performance of different electrode configurations to improve gas production efficiency. The results provide important information about how to increase the energy yield of electrolysis systems, reduce the consumption energy and develop sustainable gas production technologies.

## 2. Methodology

### 2.1. HHO wet electrolyzer components

Electrodes are used in the wet cell electrolyzer to generate oxygen at the anode and hydrogen at the cathode, respectively. Electrodes in wet cell electrolyzer are immersed in an electrolyte liquid-filled container. There is no electrolyte circulation inside the cell since the electrodes are separated from one another. Wet cells are widely used in lead-acid batteries and other battery types. In an electrolyte solution, the electrolyte only fills the gaps between them. Since the entire system acts as an electrolyzer in this case, a reservoir is not required. Depending on how the cell will be used and the needed output volume, wet cell designs may differ in size and form. Factors, including the electrolyte content, electrode material, and electrode spacing, have impact on the cell’s performance and design. The primary parts of wet cell assembly are the electrode plates, hoses, nuts, bolts, valve, electrical wires, bubbler, and glass box. The electrode plates for the electrolysis process should be made from metallic sheets.

Wet cells provide several benefits, such as higher gas output, stability, simplicity in manufacture, and simplicity in maintenance. The wet cell’s ease of production is one of its drawbacks. The configuration is not particularly portable, which causes issues with mobility. Current loss occurs as a result of the exposed electrodes on the plates that have been submerged in the electrolyte. The path that offers the least amount of resistance is used by the current when it exits the plates’ edges [40]. In addition, the wet cell exhibits corrosion at the positive electrodes, greater current need, and increased heat production from the cells. Frequently, the wet cell produces enough heat to turn the water into steam. Where the electrodes are submerged in the electrolyte, losses and corrosion take place. Wet cell electrolyzers furthermore have a propensity to overheat when in use, which is considered to be a significant drawback for practical applications of these kinds of electrolytic cells [41]. Alkaline electrolysis cell designs come in two varieties: mono-polar and bi-polar. Alternating electrodes in monopolar arrangement are parallel-connected to the DC power source’s opposing terminals. The electrolyte solution as a result contains a wide diversity of distinct cells [42]. Due to the parallel connection, which matches the voltage of the DC power supply, each cell is exposed to an equal voltage. The electrodes at the two ends of bi-polar configuration are linked to the DC power source. The electrolyte fluid connects all the other electrodes, and when they operate together, the nearby electrodes form independent cells. Since they are connected in series, the same current travels through each of these cells [42]. This design produces hydrogen and oxygen through two independent reactions on the opposing surfaces of each electrode.

**Fig.1:**
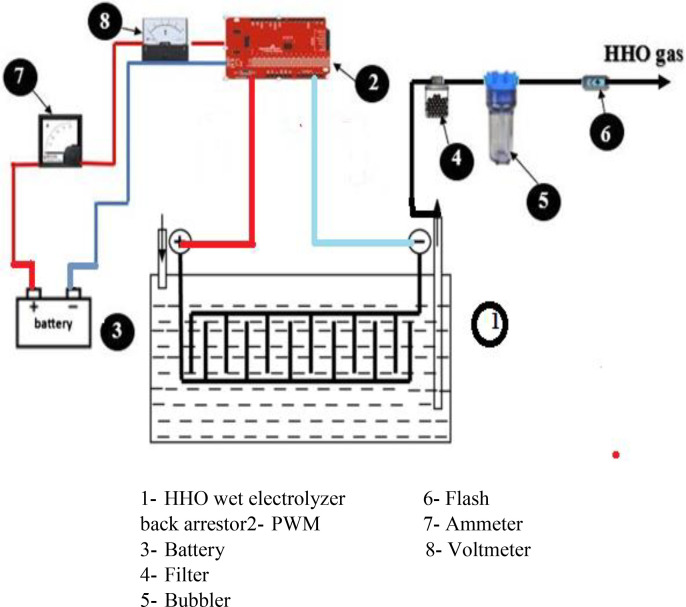
Wet electrolyzer schematic diagram.

The battery power source provided electricity at a rate of 12 VDC and 60 Amps. For the power monitoring, analogue panel ammeter and voltammeter were employed. A flow meter was applied to determine the gas volume flow rate. The electrolyzer didn’t explode because of the bubbler’s backfire. A check valve was positioned between the bubbler and electrolyzer to stop the water from the bubbler from being forced back into the electrolyzer in the case of backfire. While the HHO cell was functioning, flashback arrestors were utilized to reverse the gas supply direction and protect the system from damage and explosions. There is no physical connection between the battery and the electrolyzer cell. Instead, it is powered by an electrical relay. The electrolyzer is instantly shut off via the relay connection. A reversible circuit breaker then controls the electrical supply to the electrolyzer to avoid the increase in current and cell overheating. Pulse signals are generated by type of modulation called pulse width modulator (PWM). Since cold water cannot transmit electricity, winter water requires additional electrolytes. But during the summer, hot water carries energy more effectively than cold, therefore less electrolytes are needed in the water. The supplied current is provided during the water overheating is controlled by PWM. Fig 1 is a schematic representation of a wet cell electrolyzer.

**Fig 2:**
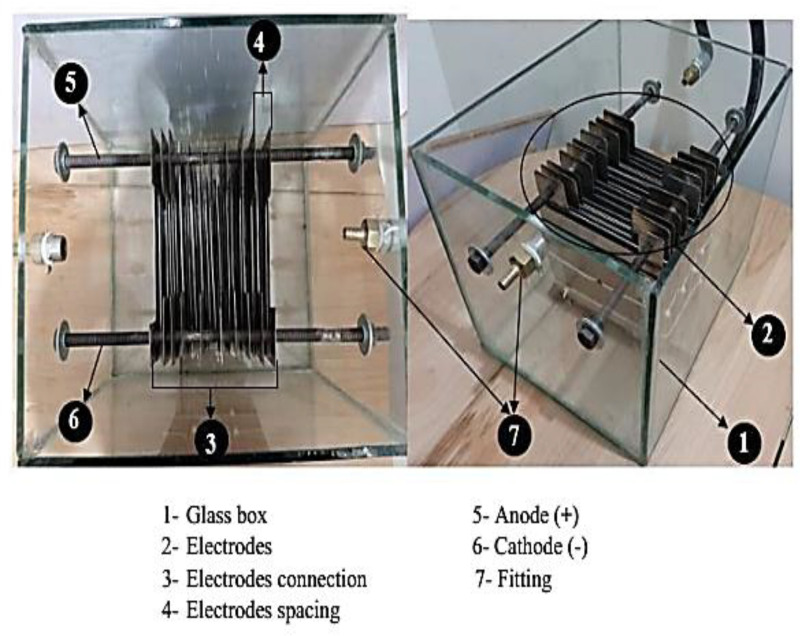
Photographic view of mono-polar wet electrolyzer [10].

Most electrons in the electric current between the electrolyte and the plates move in close proximity to the stainless steel plates since that’s the fastest path to the negative plate. Stainless steel grade 316L plates of 26 plates with area of 130 × 105 mm and thickness of 1 mm were used as electrodes. The generator’s cathode and anode are composed of the same material. The electrodes are separated by 4 mm. The input voltage is 12 VDC and 60 Ahr battery is used. The electrodes are submerged in 30 x 30 x 40 mm glass container with 10 mm thickness. Photographic view of wet electrolyzer is shown in Fig 2. Electricity is transferred from the anode to the cathode by electrolyte. The glass box that holds the plates is built using the required piping and connectors. The cell’s inputs are sodium hydroxide, which serves as an electrolyte, and distilled water.

This is due to the fact that these engine speeds are typical in conditions of moderate load, in which the engine is neither idling nor operating at maximum capacity. Since the HHO gas should meet the engine’s need for combustion improvement at these mid-range speeds (2000–3000 rpm), the gas flow rate is crucial in determining the required electrolysis cell area. The Faraday law of electrolysis, which links the current and the volume of gas is used to determine how much gas is produced. A system that can instantly modify the amount of HHO being produced in order to meet the engine’s fluctuating demands is necessary to maintain variable flow rate of HHO in response to variable-speed engine. Because the engine speed varies while operating, the HHO flow rate should be dynamically controlled to guarantee constant, ideal combustion improvement. The controller can ascertain the engine speed at any given time by equipping the engine with RPM sensors. The system may adjust the HHO flow rate in response to changes in engine power requirements, which usually increase with engine speed.

Compared to smaller cell area with fewer plates, the use of 20 plates increases the electrodes’ total surface area (assuming each plate functions as an electrode), which results in higher output of gas. Increased surface area enables the cell to effectively manage greater currents and boosting the HHO gas flow rate to satisfy the engine’s demands [7]. More current can be handled by cell with more plates such as 20 plates. By distributing the current more evenly across several plates, the electrolysis process lowers the possibility of overheating and increases cell efficiency [10]. Twenty plates could be the perfect ratio for enough gas production, current supply and compact construction [12]. KOH is more conductivity than NaOH. When it comes to electrode corrosion, NaOH is typically less aggressive than KOH. In general, NaOH gives the electrodes more stable environment for longer periods of time, which can help the equipment last longer [3,4]. Compared to KOH, NaOH is thought to be marginally less expensive and harmful. In this work, NaOH is used as a catalyst [11].

### 2.2. Electrolyzer cell configuration

The gas yield is affected by a number of variables, type, concentration of electrolyte, voltage, applied current, production temperature, production time, and plate count. The new design aims to produce more HHO with the least number of plates, electrolyte concentration, voltage, and current consumption. For the cell to work at its best, heat addition and electrical energy, or w = ∆G (Gibbs function change), are required, Q = T∆S [26,35]. Therefore,


$$\begin{array}{c} {\text{E}}_{\text{heat\textbackslash{} addition}}\;=\;\Delta \text{EQ }=\;\frac{\text{Q}}{\text{n}\times \text{ F}}=\;\frac{\text{T}\Delta \text{S}}{\text{n }\times \text{ F}} \end{array}$$ (1)

Where F is the Faraday’s constant and number of electrons is called n that has changed. Normally, the potential rises due to the thermal potential (heat addition) ∆E_Q_ [27,31]:


$$\begin{array}{c} {\text{E}}_{\text{th}}=\;{\text{E}}_{\text{rev}}+\;{\Delta \text{E}}_{\text{Q}} \end{array}$$ (2)

$$\begin{array}{c} \text{U }=\;{\text{E}}_{\text{th}}\;+\text{ E} \end{array}$$ (3)

Where U is the necessary voltage difference that used to keep electrolysis from overheating. Nevertheless, E stands for the potential variation needed to overcome the resistance of electrolyte [35].


$$\begin{array}{c} \text{E }=\text{ IR,\textbackslash{} i }=\;I/A\; \end{array}$$ (4)

$$\begin{array}{c} RA=\frac{Gasket\;thickness\;(separation)}{Electric\;conductivity\;of\;water} \end{array}$$ (5)

The symbols R and I stand for the resistance of electrolyte and current density, respectively. The two plates’ contact resistance (RA) is displayed. The used electrolytes are solutions of NaOH and KOH. Tap water is utilized to decrease the electrolyzer corrosion [43]. At 25% concentration, the electric conductivity of water is 520 mS/cm. The spacing between the plates inside the cell varies from 1 to 9 mm. Between the two plates, sodium hydroxide electrolyte has contact resistance (RA) of 769 cm^2^. Stainless steel 316L is a good material for electrodes because it has better mechanical characteristics and is more corrosion resistant. Faraday showed that 0.54 Amp can be evenly dispersed across the 1 square inch of surface area of a plate [26,35]. As a result, each pair of stacks should have total voltage of at least 1.54V. One mole of hydroxy and two moles of electrons were sent for each mole of used water. Here are a few instances of how the current directly impacts the rate of oxyhydrogen gas production [26]:


$$\begin{array}{c} \frac{{dN}_{HHO}}{dt.A}-\frac{i}{n\times F}\; \end{array}$$ (6)

Where (dN_HHO_/dt.A) represents the hydroxy rate per single plate cross section area. The designed HHO generator was used with 200 cm^3^ sweeping volume engines [28,32].


$$\begin{array}{c} \frac{{dN}_{HHO}}{dt}-\frac{PV_{s}^{o}\;}{R_{u}T} \end{array}$$ (7)

Where dN_HHO_/dt denotes the HHO production rate. On the other hand, V^0^s is the piston’s swept volume. The idea gas equation was used to get the HHO generation rate in moles per minute. The engine used a 20% HHO volume percentage and ran on dual fuel [29,33].


$$\begin{array}{c} V_{HHO}^{o}\;=\;X_{HHO}\;\times \;V_{fuel}^{o}\;=\;1.6\;L/min \end{array}$$ (8)

$$\begin{array}{c} N_{HHO}^{o}=\;\frac{P\times V_{HHO}^{o}}{R_{u}\times T}=\;0.065\;moles/min \end{array}$$ (9)

$$\begin{array}{c} \frac{\frac{{dN}_{HHO}}{dt}}{\frac{{dN}_{HHO}}{dt.A}}=3495\;{cm}^{2} \end{array}$$ (10)

With suitable cross section area of 1310.5 cm^2^, each plate can be divided. The input current and voltage ranges are from 6 to 42 A and from 2 to 11 volt, respectively, depending on the number of stacks and temperature. The battery voltage varies from 1.53 V to 12 V. The ratio of energy supplied to energy applied to run the system is known as the generator efficiency. One useful byproduct of water electrolysis that is created when water breaks down is HHO gas. This reaction is endothermic, meaning it needs 285.85 kJ/mol of energy to complete. The moles of gas under STP conditions are predicted by the ideal gas equation [38]. The amount of energy supplied to the electrolysis process is determined by the input voltage and current. Several metrics used to measure the efficiency of an electrolyzer are based on the percentage of the total voltage used for electrolysis [44]:


$$\begin{array}{c} \eta_{Voltage}=\frac{E_{anode}-E_{cathode}}{V_{cell}}\; \end{array}$$ (11)

The theoretical required energy and process energy losses are used to compute the Faradic and thermal efficiencies of electrolysis [31,34]:


$$\begin{array}{c} \eta_{\text{Faradic}}=\frac{\Delta \text{G}}{\Delta \text{G + losses}}=\;\frac{{\text{E}}_{\Delta \text{G}}}{{\text{V}}_{\text{cell}}} \end{array}$$ (12)

$$\begin{array}{c} \eta_{\text{thermal}}=\frac{\Delta \text{H}}{\Delta \text{G}+\text{losses}}-\frac{{\text{V}}_{\text{th}}}{{\text{V}}_{\text{cell}}} \end{array}$$ (13)

$$\begin{array}{c} \eta_{net\;efficiency}=1-\frac{E_{loss}}{E_{input}} \end{array}$$ (14)

The following formula was used to determine the experiment’s overall uncertainty.


$$\mathrm{\backslash [}\sqrt{(uT)2+\;(ut)2\;+\;(uV)2\;+\;(uA)2\;+\;(uC)2\;+\;(uQ)2}=\sqrt{(1)2\;+\;(1.5)2\;+\;(0.8)2\;+\;(1)2\;+\;(0.5)2\;\;\;\;}=\pm 2\;\%\mathrm{\backslash ]}$$

uT stands for electrolyte temperature uncertainty, uv for applied voltage uncertainty, uA for applied current uncertainty, ut for operating time uncertainty, uC for electrolyte concentration uncertainty, and uQ for gas volume rate uncertainty as shown in Table 1.

**Table 1:** Accuracy, ranges, and uncertainty of measured parameters.

No.	Device	Range	Accuracy	Uncertainty
**1**	**Voltammeter**	**0-30 Volt**	**±1 Volt**	**±3%**
**2**	**Electronic balance**	**0-1000 mg**	**±1 mg**	**±0.1%**
**3**	**Ammeter**	**1-100 Amp.**	**±1 Amp.**	**±1%**
**4**	**Temperature thermocouple K**	**0-100 °C**	**±1°C**	**±1%**
	**Catalyst concentration**	**0-20%**	**±1%**	**±1%**
**5**	**Timer**	**0-120 sec.**	**± 1 sec.**	**±0.85%**
**6**	**Gas flowmeter**	**0-5000 ml/min**	**±50 ml/min**	**±1%**

**Fig 3:**
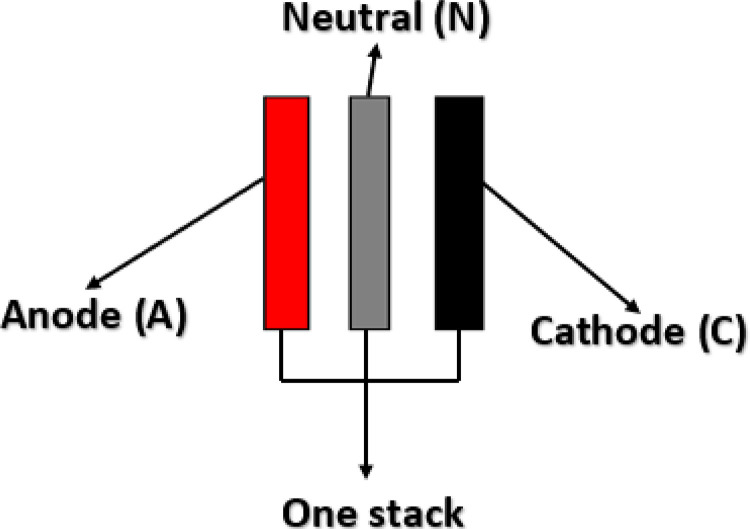
Electrodes connection.

To produce the peak gas production, the plate configuration is examined. As shown in Fig 3, the neutral, anode, and cathode plates are designated C, A, and N, respectively. Red, black, and grey electrode connectors stand for the positive, negative, and neutral electrode connections, respectively. A single stack is represented by each set of red, black, and gray poles. Cathode, anode, and neutral electrode configurations as indicated in Table 2 are taken into account when rearranging the electrodes into various combinations.

**Table 2:** Configurations of electrodes.

Test	Configuration	Cathode number	Anode number	Neutral number
**1**	**3C3A0N**	**3**	**3**	**0**
**2**	**3C3A2N**	**3**	**3**	**2**
**3**	**2C1A8N**	**2**	**1**	**8**
**4**	**7C7A0N**	**7**	**7**	**0**
**5**	**7C7A2N**	**7**	**7**	**2**
**6**	**7C7A4N**	**7**	**7**	**4**
**7**	**5C5A10N**	**5**	**5**	**10**
**8**	**6C5A10N**	**6**	**5**	**10**
**9**	**10C10A5N**	**10**	**10**	**5**
**10**	**4C3A19N**	**4**	**3**	**19**
**11**	**6C5A15N**	**6**	**5**	**15**

The effects of neutral plates and electrode configurations on HHO production were demonstrated. It was investigated how different catalyst types, such as NaOH and KOH, affected the rate of hydroxy gas. Impact of catalyst concentrations variations (0–20 g/L) on hydroxy gas production was shown. The effect of voltage fluctuations between 2 and 11 volts on the yield and efficiency of HHO generators was investigated. It was examined how changes in current from 6 to 42 Ampere affected the efficiency and yield of HHO generators. The impact of operating times ranging from 0 to 120 minutes on gas output was examined. The effects of varying cell gaps of 1, 3, 4, and 7 mm on the flow rate of oxyhydrogen were examined. As electrolytes, NaOH and KOH improve water’s conductivity and lower electrical resistance. The electrolyte solution may have low conductivity at less than 5 g/L, which would result in poor current flow, increased resistance, and decreased efficiency of HHO synthesis. The conductivity gains decrease and other problems, including excessive heat generation or electrode deterioration, become more serious at concentrations more than 20 g/L. The catalyst percentage range of 0–20 g/L is chosen because it balances the relationship between the amount of HHO produced and applied current. Too high catalyst concentrations can increase energy losses and high concentrations of NaOH are highly corrosive to electrodes. There is less chance of the solution overheating, which could cause thermal instability or damage to the equipment. This range is a practical balance between conductivity, efficiency, and safety, making it an ideal choice for HHO production. All of the electrodes (anodes and cathodes) in mono-polar design are linked in parallel to the power source’s electrical poles. A constant voltage is delivered to every pair of electrodes, allowing each pair to function independently. In order to promote electrolysis, mono-polar design needs low supply voltage and demands large current. The electrolyzer design is mono- polar.

## 3. Results and discussions.

### 3.1. Impact of electrodes configuration on oxyhydrogen flow rate

The electrode designs of wet cells at various NaOH ratios are shown in Table 3 shows variable electric current and constant 6.25 V voltage. Wet cell design [3C3A0N] is not advised due its poor HHO gas generation and higher current consumption. Because the produced gas is insufficient for engines with lower power, it is not recommended. Although the designs [3C3A2N] and [2C1A8N] are utilized to decline the current, they are not recommended because of its lower rate. It is also not recommended to use wet cell design [7C7A0N] because of the higher current used, low catalyst percentage, and less gas yield. It is not recommended to utilize configuration [7C7A2N] since it requires higher current and catalyst ratio. Due to the greater voltage source requirement and increased of current leakage, [5C5A10N] is not recommended for usage. Additionally, using configuration [6C5A10N] is not recommended, which requires higher concentration of NaOH, more current, and lower generation of gas. The surface area, active response and current were increased with the number of plate’s increases. When the number of electrodes increases, current flow increases as well, and when the electrode gap widens, current flow decreases. Because it produces less gas rate and necessitates higher catalyst ratio and current, configuration [10C10A5N] should not be employed. Due to the significant enhancement in gas yield, configuration [4C3A19N] is not recommended for wet cells with the consideration of plate’s number, current and electrolyte concentration. Raising the NaOH level increases the gas rate and electrical conductivity. Wet cells are best used with configuration [6C5A15N], as adding electrodes increases overall surface area and response activity. Water dissociation and electron transport between the electrodes both increase as current increases. The oxyhydrogen generator’s effective surface area for oxidation and reduction reactions is reduced when the number of electrodes (anode and cathode) is reduced. This, in turn, lowers the amount of produced brown gas. Thus, increasing the number of electrodes may improve electrolysis’s efficiency. The neutral plates promote the production of gas by the potential reduction between plates. Provided current is reduced and the effective area is improved by the neutral plates. This is due to the fact that as the number of electrodes grows, so does the surface area contact for the electrolysis process. The amount of brown gas generated rises but the current falls with the number of neutral plates between the anode and cathode electrodes. Temperature is raised by the potential decrease, which also promotes the successful collisions, increases ionic mobility, and significantly increases the production of HHO gas. Neutral electrodes placed between the cathodes and anodes improve the effective area while lower the electric current without altering flow rate [46,47].

**Table 3:** Cell configurations with constant voltage and different current and NaOH concentrations.

Test	Configuration	Current, Amp.	Plates number	Flow rate, ml/min	NaOH concentration
**1**	**3C3A0N**	**9**	**6**	**135**	**10%**
**2**	**3C3A2N**	**12**	**8**	**195**	**20%**
**3**	**2C1A8N**	**18**	**11**	**295**	**20%**
**4**	**7C7A0N**	**25**	**14**	**325**	**10%**
**5**	**7C7A2N**	**23**	**16**	**377**	**20%**
**6**	**7C7A4N**	**32**	**18**	**584**	**20%**
**7**	**5C5A10N**	**28**	**20**	**630**	**5%**
**8**	**6C5A10N**	**30**	**21**	**825**	**10%**
**9**	**6C5A10N**	**34**	**21**	**914**	**20%**
**10**	**6C5A10N**	**39**	**21**	**815**	**30%**
**11**	**10C10A5N**	**48**	**25**	**1145**	**10%**
**12**	**10C10A5N**	**60**	**25**	**1376**	**20%**
**13**	**4C3A19N**	**7**	**26**	**695**	**5%**
**14**	**4C3A19N**	**10**	**26**	**743**	**10%**
**15**	**4C3A19N**	**15**	**26**	**850**	**15%**

### 3.2. Electrolyte type effect on HHO flow rate

The Na+ cation has lower potential than the hydrogen electrode ions. During the electrolysis process (oxidation), an ion in the electrolyte competes with hydroxide to liberate an electron. Ions have lower potential electrodes than hydroxide ions. Cations have higher electrode voltage about hydrogen ions due to the reduction process. Different electrolytes, currents, and designs are conducted at constant voltage of 6.25 V, as indicated in Table 4. Hydroxy gas rate using 20 plates and 12 Ampere is 275 ml/min [8C8A4N]. KOH at 16 A and 20 plates produces 285 ml/min flow rate for wet cell [8C8A4N]. Distilled water is needed to maximize the use of sodium hydroxide. Without an electrolyte, tap water loses some of its electrical conductivity. As electrolyte content increases, the conductivity and ion collisions were enhanced. The rate of water dissociation was impacted by the increase in electrolyte concentration because it improved the conductance of the electrodes and the rate of electron transfer between them. This, in turn, increased the electric current. While the decrease in electrolyte resistance results in the increase in electrical conductivity and possible reduction in electrical resistance, increase in concentration lowers electrical resistance and increases electric current. Higher electrolyte concentrations speed up molecular motion in the water, resulting in more forceful collisions between oxygen and hydrogen molecules. The kinetic energy between molecules rises with the intensity of the molecule-to-molecule collision, increasing the amount of produced HHO gas. The current and gas production were decreased using tap water due to the increased resistance. Potassium hydroxide is recommended with the same structure and electrolyte content since it has lower molecular weight than other solutions. These results are supported by the studies mentioned [31,35].

**Table 4:** Impact of electrolyte on production rate of HHO.

Plates No.	Connection	Electrolyte	Current, Amp.	Flow rate ml/min
**20**	**8C8A4N**	**Tap water**	**10**	**215**
**20**	**10C10A0N**	**Tap water**	**13**	**187**
**20**	**8C8A4N**	**1 g NaOH/L**	**12**	**275**
**20**	**8C8A4N**	**1 g KOH/L**	**16**	**285**
**20**	**10C10A0N**	**1 g NaOH/L**	**18**	**248**
**20**	**10C10A0N**	**1 g KOH/L**	**20**	**255**

### 3.3. Influence of voltage on oxyhydrogen rate

**Fig 4:**
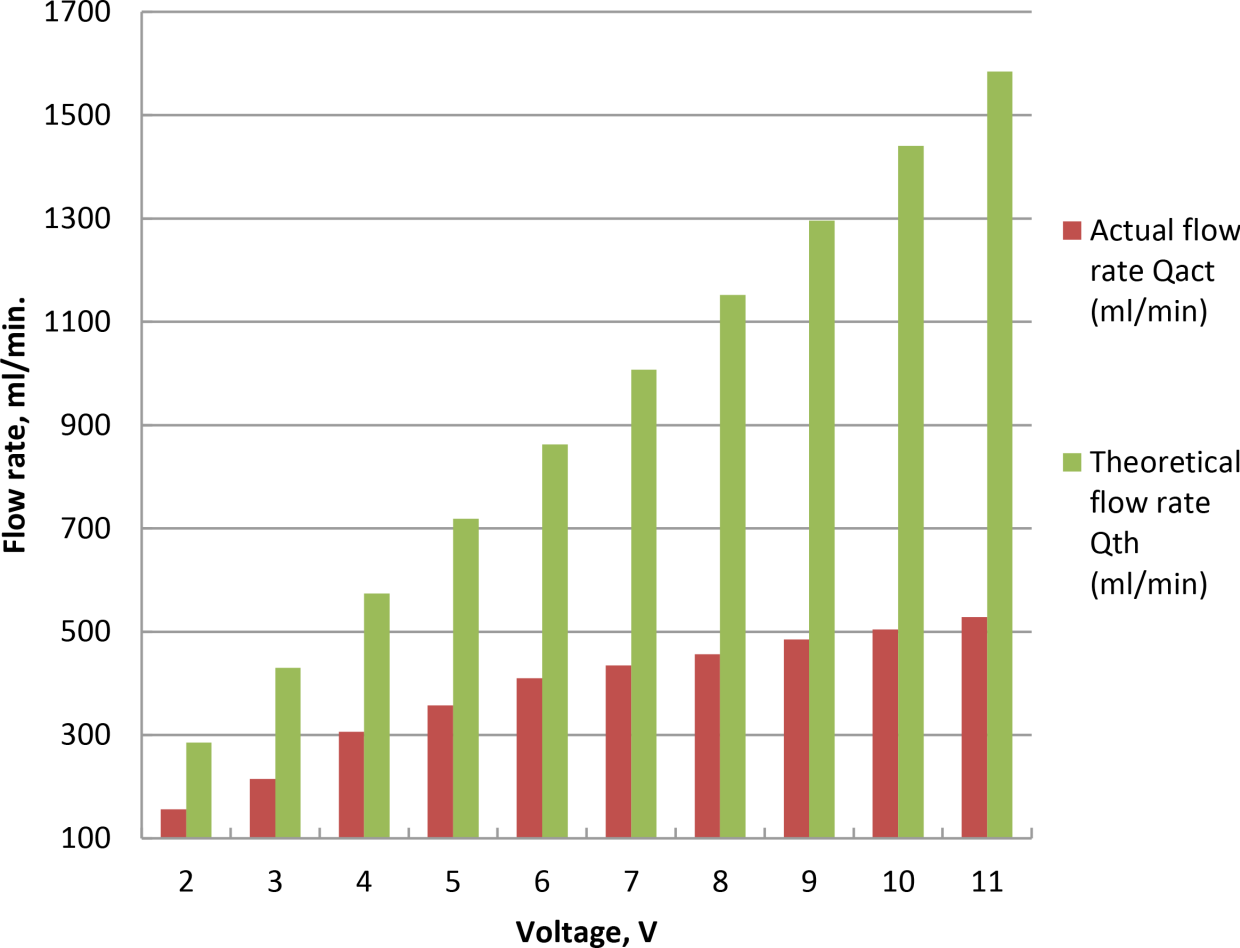
Voltage fluctuation effect on HHO rate at concentration of 5% NaOH.

**Fig 5:**
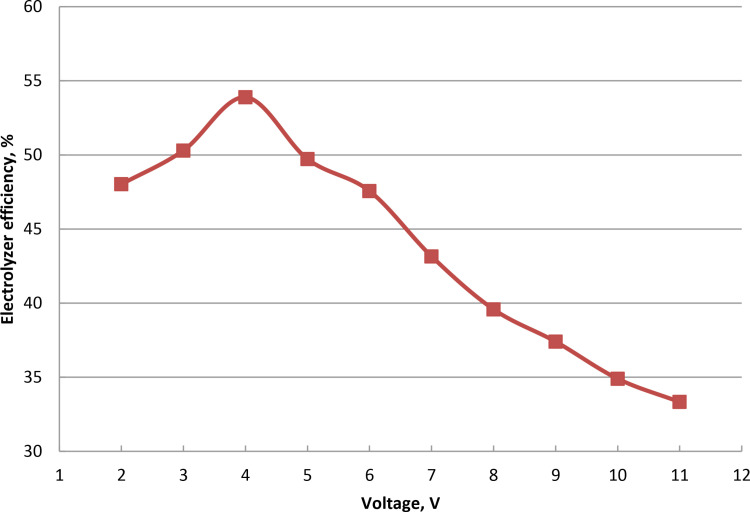
Electrolyzer efficiency at different voltage at 5% NaOH ratio.

The applied voltage and current in wet cell determine how quickly HHO is produced. Gas generation is examined over electrodes at 25°C room temperature and 5% catalyst ratio. The voltage was changed from 2 to 11 volt while keeping the current at 7 Ampere constant, as indicated in Fig 4 and Supporting Information. To start the electrolysis process, minimum voltage known as the decomposition or threshold voltage is needed, which roughly 1.23 V per cell for water is splitting under normal circumstances. After the threshold voltage is reached, additional voltage accelerates the synthesis of HHO by increasing the system’s current. Because of the increased current flow, higher voltages can cause the electrolyte to generate a considerable amount of heat. Elevated voltages can hasten electrode corrosion and wear. As the voltage rises, the generation rate rises steadily due to the acceleration of reaction kinetics, uniform charge density, and ion exchange on the electrode. Flow rate of 528 milliliters per minute at 11 Amps is the peak. This may be because the exchange factor between the electrons and positively charged hydrogen ions increased as the current increased. The electrolyzer efficiency was raised to 51.12% for wet cell when the potential was raised from 2 to 4 volt. The efficiency decreases at voltages increase between 5 and 11 V, as seen in Fig 5 and S3 Table in S1 File. This is brought on by the increase in real flow rate that is approximately theoretical up to 5 V, after which a fall in actual flow rate is brought on by the increase in current leakage. Higher voltage causes higher current, which raises the electrolyte’s temperature and reduces the efficiency even further. Lower efficiency and higher energy losses were shown because more energy is lost as heat. Temperature rises brought on the cell overvoltage reduce the possibility for gas production. The cell’s efficiency drops as the result of overvoltage. Production of HHO gas is improved as the result of potential growth. This result is consistent with the findings of the cited literature [46,48].

### 3.4. Effect of applied current on hydroxy rate

**Fig 6:**
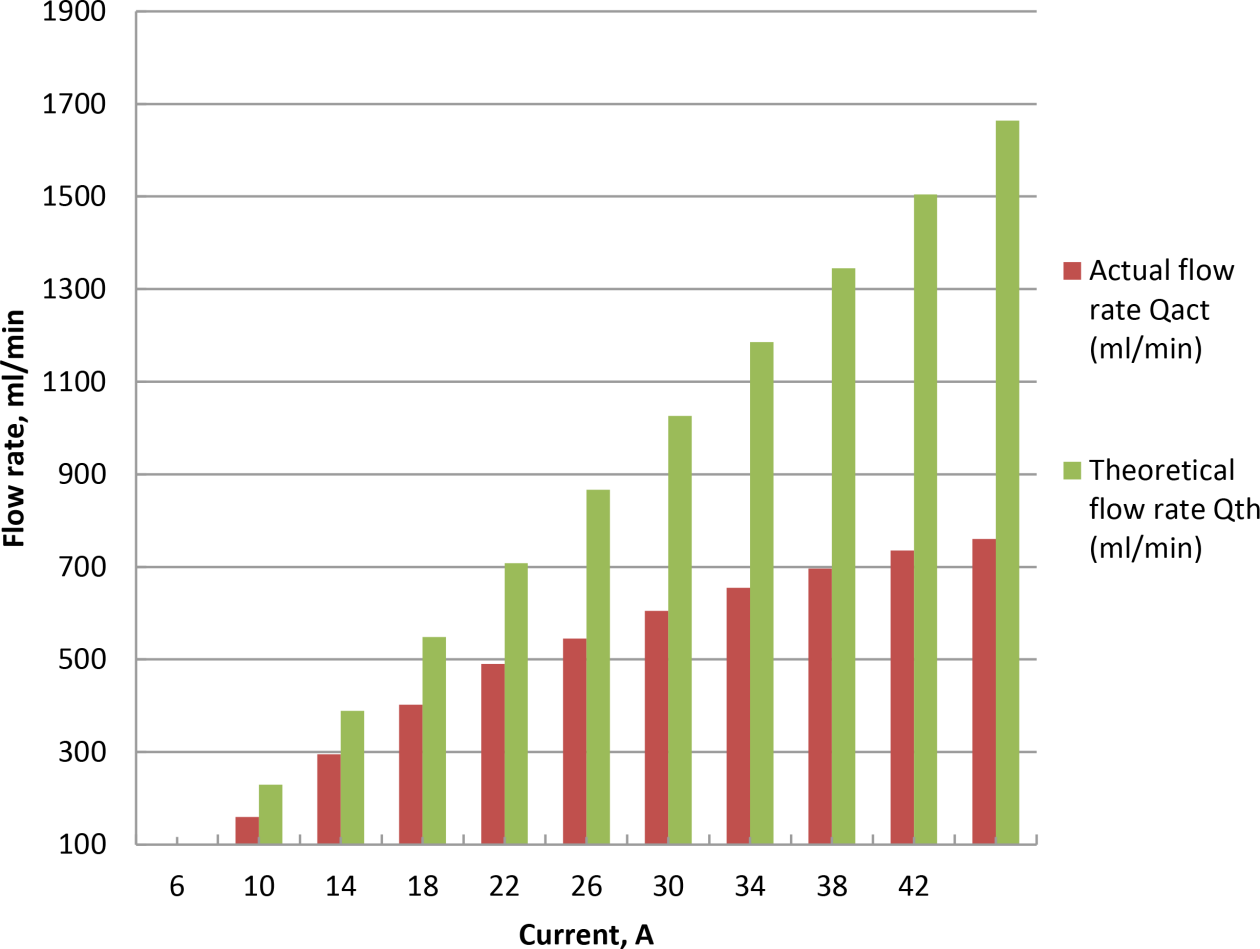
Influence of current variation on oxyhydrogen using 5% NaOH concentration.

**Fig 7:**
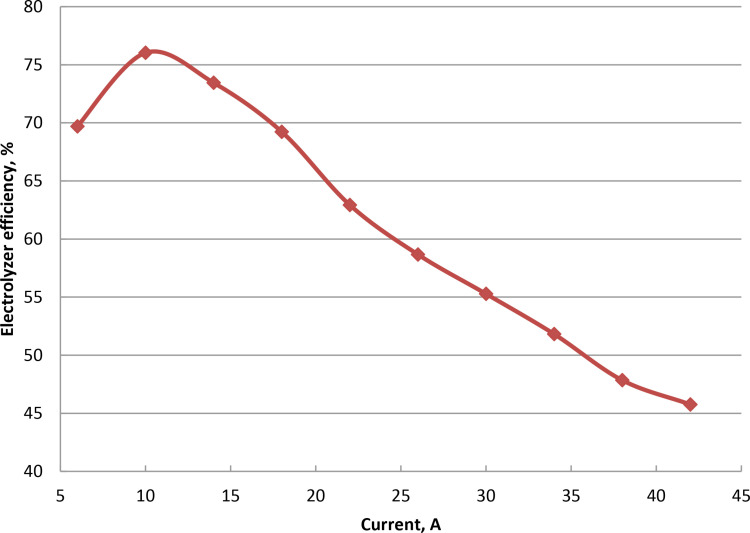
Electrolyzer efficiency at different currents in 5% NaOH concentration.

As illustrated in Fig 6 and Supporting Information, the applied current can be adjusted between 6 and 42 Amp while maintaining a constant voltage. As the applied current increases, the rate of gas production in wet cell progressively increases. Higher applied current results from bubbles forming on the electrode surface more quickly. When 14 Amp current is used by the oxyhydrogen generator, bubbles form. Electric current flow is impacted by the electrode instability brought on by the produced bubbles. Electron exchange between hydrogen ions and the electrons increases with the applied current. The current increase results in the effective ion collisions and increases the conductance of the electrode. As current increases, electrons flow between electrodes more quickly. The amount of dissociation of water is influenced by the transferred electrons. Produced heat in the electrolyte by excessive currents results in energy losses and may lower the efficiency of gas production. The present increase results in less efficient plate heating and water evaporation. The maximum HHO flow rate at 42 Amp is 760 ml/min. Excessive current wastes energy, over potential losses and decreases efficiency by heating the electrolyte excessively. The electrolyzer’s efficiency increases to 75.45% when the current is increased from 6 to 14 Amp. But after that, as in Fig 7 and S4 Table in S1 File, the electrolyte replenishing process is reduced, leading to a drop in efficiency. These results were agreed with references [7,10,48].

### 3.5. Effect of operating time on current and oxyhydrogen production rate

**Fig 8:**
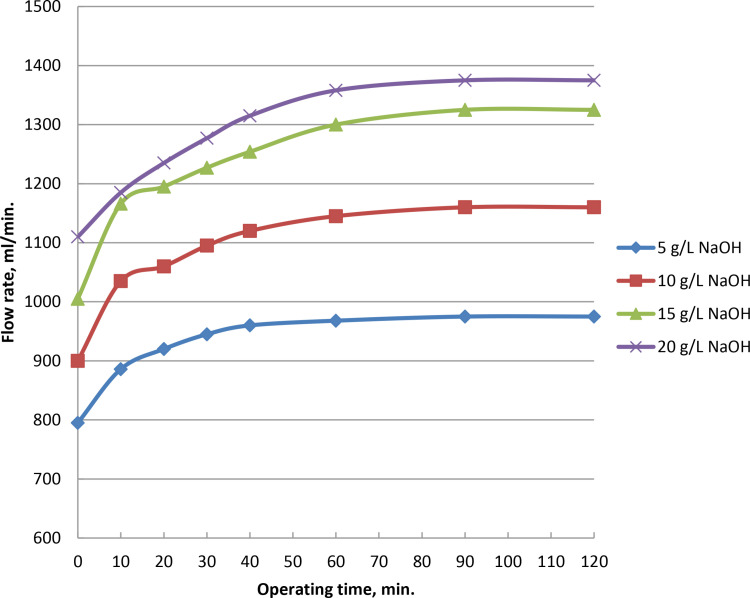
Impact of time of operation on gas rate at NaOH variations.

**Fig 9:**
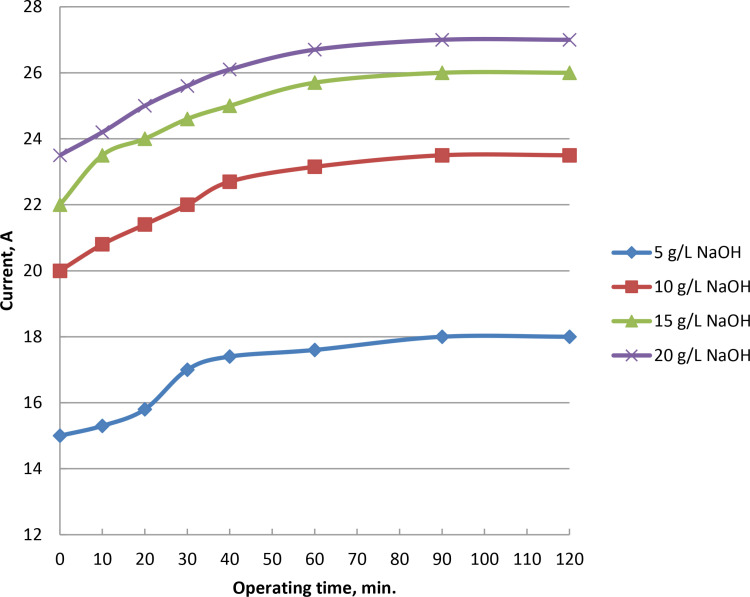
Influence of operating time and NaOH concentration variation on electrolyzer current.

As the electrolysis begins, gas generation grows steadily. At 5, 10, 15, and 20 g/L of catalyst ratios, the rate of gas production reach to peaks at 975, 1160, 1325, and 1375 ml/min, respectively, after 90 minutes. Fig 8 and Supporting Information demonstrate that wet cell with 5 g/L sodium hydroxide catalyst may generate the highest HHO gas at yield of 975 ml/min when the voltage is kept at 6.25 V. Wet cell electrolysis takes 90 minutes, at which point the electrode is stable and doesn’t dirty the active surface. The average kinetic energy of their constituent molecules increased as the result of slow rise in movement between the molecules of hydrogen and oxygen gases created during the electrolysis process. As a result, there were more effective molecular collisions per unit of time, which raised the observed output of HHO gas. A respectable level of stability is the outcome of the stainless steel electrode’s performance. The frequency of collisions and internal movement between the molecules of oxygen and hydrogen increases are associated with the time of production process. As a result, more hydroxy gas is created and the average kinetic energy of the molecules rises. Current increases and electrical resistance decreases in proportion to the increase in electrolyte content. Electrical conductivity rises and potential falls with decreasing electrolyte resistance. Electrode conductivity and electron transport are enhanced by the increases in current and NaOH concentration as in Fig 9 and S5 Table in S1 File. With an electrolyte concentration of 5, 10, 15, and 20 g/L of catalyst and currents of 17.8, 23.5, 26, and 27 Ampere, as shown in Fig 9, the highest gas rate is reached after around 90 minutes.

### 3.6. Impact of operating time on electrolyte temperature

**Fig 10:**
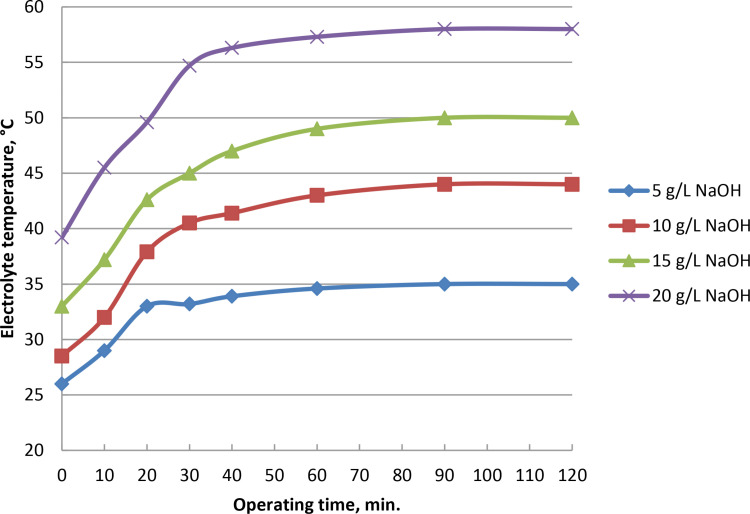
Effect of NaOH concentrations and electrolyte temperature on production of HHO gas.

Production of hydroxy gas during the electrolysis process increases with temperature during the wet cell’s operation period. At a concentration of 5 grams of NaOH electrolyte, the temperature was increased to 35°C after 32 minutes. It remains there as long as the electrolysis procedure is reliable and fouling-free. The increase of temperature increases is due to the heat transfer increase on the plates and mobility of electrons. At catalyst concentrations of 10, 15, and 20 g/L, the temperatures were increased to 44, 50, and 58°C, respectively, and remain steady for 32 minutes. Fig 10 and Supporting Information show how operation time affects the electrolyte temperature at different catalyst percentages at voltage of 6.25 V and fixed current of 17.8 Amp. The production temperature rose when the HHO gas was produced on time. As a result, the generator was more effective and used less energy throughout manufacturing. Additionally, the slow rise in temperature enhanced the electrolyte’s ionic conductivity, and improved the electrodes’ surface reaction. Rise of production temperature increases the conductivity of ions and electrodes surface responsiveness while decreasing the rupture potential of water molecules. The electrolyte’s electrical resistance and potential both decrease with operating temperature increase. Electrolyte heated as the result of resistance between the electrodes and substance. The molecule collisions lead to the temperature rise, more ions were produced and more creation of gas. Both water evaporation and electrolyte concentration increase as the electrolyte temperature rises. Prior to reaching working conditions, the lowest feasible electrolyte concentration must be achieved. Rapid reaction kinetics and explosives are the outcomes of the temperature rise. The outcomes align with previous studies [47,48].

### 3.7. Cell gap impact on gas rate

**Fig 11:**
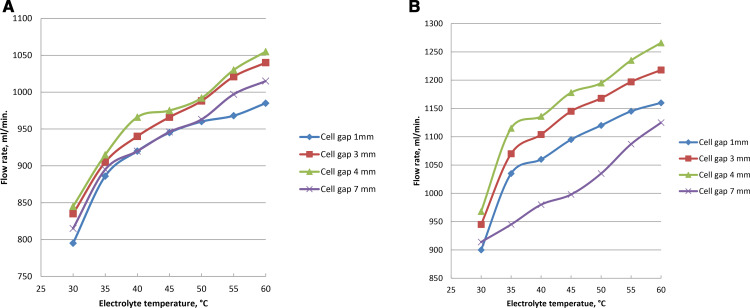
(a): Impact of electrolyte temperature on gas formation at 5% NaOH with cell spacing variation. (b): Gas rate at 10% NaOH with temperatures of electrolyte and cell gaps variations.

The rate of gas increases for the same gap of cell when the electrolyte ratio rises in each cell. Production rate increases as the cell gap increases at 1, 3, and 4 mm in wet type. The formation of HHO of wet cell reduces at cell gap of 7 mm as the electrolyte temperature rises from 30 to 60 ^0^C. The procedure needs a lot of cooling water and a low operating temperature because of the tiny cell gap. Figs 11(a), (b) and Supporting Information display the gas rates at cell distances (1, 3, 4, and 7 mm) at 5 and 10% catalyst concentration. Temperature caused a decrease in the energy potential needed to break water molecules. The flow rate of brown gas rises as the electrode gap gets smaller. This is due to the fact that resistance increases with the distance between the two electrodes. Therefore, the resistance can be reduced by reducing the distance. The shortest path should be followed to keep the gap from closing because reducing the distance between the electrodes lowers resistance. The operational current of oxyhydrogen generator is likewise impacted in such a way that current rises as the gap closes. A distance that is too small, however, is inappropriate since it affects the temperature because of the rise in current. On the other hand, the electrolyte’s surface response at the active sites and ionic conductivity both increased with temperature. The increase of cell gap leads to the improvement in the rate of hydroxy and the boiling temperature of the electrolyte between the poles was decreased. For the wet cell, 4 mm cell gap is therefore ideal. Three to four millimeters between cells is the appropriate spacing for HHO generator. The bubbles expand and obstruct the active reaction sites when the electrolyte’s temperature rises. With larger cell gap, the gas bubbles should be able to exit the cell and detach from the surface of cell faster.

### 3.7. Recommended electrolyzer cell configuration

When the electrodes are connected and the electrolyte percentage is appropriate, the wet cell receives a minimum current of 16–22 Ampere. At the lowest feasible voltage, operating temperature, current consumption, and working duration, the maximum HHO flow rate is offered. [6C5A15N] is the recommended cell setup connection. Wet cell electrode spacing is 4 mm, and the electrolyzer efficiency is 69.1%. At current of 17.8 Amp, the wet cell electrodes produce 1160 ml/min of oxyhydrogen gas. There is frequently enough heat produced in the wet cell to turn the water into steam. At the locations where the electrodes are submerged in electrolyte, losses and corrosion take place. Additionally, wet cell electrolyzers have a propensity to overheat while in operation, which is seen to be a significant drawback for practical applications of these kinds of electrolytic cells. Table 5 displays the approximate cost of an HHO wet cell.

**Table 5:** HHO production cell estimated cost.

Generator parts	Evaluation cost ($)
**Plates (13x10.5 mm**^**2**^)	**35**
**Bolts and screws**	**20**
**Connecting wires**	**10**
**Bubbler**	**10**
**Glass box (20 x 30 x10 mm**^**3**^)	**40**
**DC ammeter, voltammeter and PWM**	**100**
**Circuit breaker, Relay and fuse**	**15**
**Total cost**	**230** $

## 4. Conclusions

Wet water electrolyzer with stainless steel 316L plate electrode produced HHO gas. The electrolyte content, connections between the electrolyzer cells, electric current, operating temperature, time, and voltage were all taken into consideration when analyzing the productivity of HHO. The electrode surface area of 136.5 cm^2^ stainless steel of the cell was utilized. The following conclusions could be made based on the current investigation:

- Plates of 316 L stainless steel with six anodes, five cathodes, and fifteen neutral were used in the wet cell to produce the most HHO gas. Neutral plates between the anodes and cathodes reduced performance losses and electric current. Increase of current and voltage improves the gas generation.
- The use of tap water increased the resistance which resulted in the decreases in utilized current and hydroxy rate. The effective area was increased and supply current was decreased by the use of neutral plates. KOH is consequently better than NaOH for the same electrolyte percentage and design because of its lower molecular weight.
- HHO gas rate was increased steadily as the applied voltage was raised. The electrolyzer efficiency was improved to 69.3 with the voltage increase from 2 to 5 volt but it was decreased with the increase in voltage from 5 to 11 volt. The current rise led to the increase in gas flow rate. When increasing the current from 6 to 14 A, the efficiency of the dry electrolyzer increased to 75.45%; however, it then decreased due to a decrease in electrolyte replenishment.
- Rate of gas formation peaked at 975, 1160, 1325, and 1375 ml/min, respectively, after 90 minutes at NaOH ratios of 5, 10, 15, and 20 g/L but the currents were 17.8, 23.5, 26, and 27 Ampere, respectively. At the same concentrations of NaOH, temperatures were increased to 35, 44, 50, and 58 0C, respectively, and then kept there.
- When the cell gaps are 1, 3, and 4 mm, the production rate was increased for wet cells; however, when the electrolyte temperature increased from 30 to 60 °C, gas yield was decreased at 7 mm. Thus, 4 mm cell gap is recommended for the wet cell. The ideal cell spacing for an HHO generator is between 3 and 4 mm.
- Highest gas productivity of 975 ml/min with a production efficiency of 69.3% was achieved by operating the cell design [6C5A15N] with an applied current of 17.8 Ampere and electrolyte ratio of 10 gram per liter.
- From the literature, it was evident that the constructed dry cell performed better overall than the wet cell in all respects, including greater HHO gas evolution and lower operating temperatures.
- Since the HHO wet cell gets its energy from a battery source, it is more cost-effective than the dry cell. Wet cell electrolyzers have a propensity to overheat while operating, which is seen as a significant drawback for practical applications of these kinds of electrolytic cells.

### Future research directions

Advanced electrode materials should be investigated to boost efficiency and durability. Non corrosive alternative electrolytes can be used to provide improved conductivity. Energy recovery systems can be used to reduce power losses while producing HHO. The renewable energy sources to sustainably power the HHO production can be investigated. A control system that can regulate HHO production with engine load and speed fluctuations can be applied.

### Practical applications

HHO systems can be used in automobiles to lower emissions and increase fuel economy. HHO producing systems can use backup power sources or range extenders in electric or hybrid cars. HHO gas can be applied as an alternative fuel in boilers, furnaces, or industrial combustion operations to increase energy efficiency and lower carbon footprints.

### Drawbacks of wet electrolyzer application

Water in the electrolyte gradually evaporates as the cell works, necessitating regular replenishing to keep it functioning efficiently. Over time, deposits on the electrodes caused by impurities in the electrolyte or water, such as dust, debris, or dissolved salts, can lower performance. This leads to the corrosion of wet cell. Electrolyte leakage is a potential problem for wet HHO cells, particularly in moving systems like automobiles. Leakage can be dangerous and lead to corrosion. Long-term use or high current levels can produce a lot of heat, which lowers gas production efficiency and can harm parts. To maintain ideal operating temperatures, wet HHO cells frequently need extra cooling devices like fans or heat exchangers. The electrode surfaces may accumulate deposits of salts, minerals, or oxides, which would lower their conductivity and efficiency of gas production. It needs to be cleaned or replaced frequently. For small-scale applications, wet HHO cells are more appropriate. Due to the immersion in electrolyte solution, electrodes may corrode with time at high current densities.

## Supporting information

S1 File **S1 Table**. Effect of voltage variation on flow rate. **S2 Table**. Effect of current variation on flow rate. **S3 Table**. Effect of voltage variation on cell efficiency. **S4 Table**. Effect of current variation on cell efficiency. **S5 Table**. Effect of operating time on current. **S6 Table**. Effect of operating time on HHO flow rate. **S7 Table**. Effect of cell gap on HHO flow rate.
(PDF)
