# Correction: Examining oxyhydrogen gas generation experimentally using wet cell design

**DOI:** 10.1371/journal.pone.0339278

**Published:** 2025-12-17

**Authors:** M. S. Gad, A. K. El Soly, Subhav Singh, Kamal Sharma, Saurav Dixit, Md irfanul Haque Siddiqui

There are errors in the author affiliations. The correct affiliations are as follows:

M. S. Gad^1^, A. K. El Soly^2^, Subhav Singh^3,4,5^, Kamal Sharma^6^, Saurav Dixit^7,8^, Md irfanul Haque Siddiqui^9^

1 Mechanical Engineering Department, Faculty of Engineering, Fayoum university, Fayoum, Egypt, 2 Mechanical Engineering Department, Faculty of Engineering, Al Azhar university, Cairo, Egypt, 3 Centre for Promotion of Research, Graphic Era (Deemed to be University), Uttarakhand, Dehradun, India, 4 Chitkara Centre for Research and Development, Chitkara University, Rajpura, Himachal Pradesh, India, 5 Division of research and development, Lovely Professional University, Phagwara, Punjab, India, 6 Department of Mechanical Engineering, GLA university, Mathura, India, 7 Centre of Research Impact and Outcome, Chitkara University, Rajpura- 140417, Punjab, India, 8 Division of Research & innovation, Uttaranchal University, Dehradun, India, 9 Department of Mechanical Engineering, College of Engineering, King Saud University, Riyadh, Saudi Arabia.

## References

[1] Gad MS, El Soly AK, Singh S, Sharma K, Dixit S, Siddiqui MiH. Examining oxyhydrogen gas generation experimentally using wet cell design. PLoS One. 2025;20(6):e0324921. doi: 10.1371/journal.pone.0324921 40460324 PMC12133180

