# Supplementary material for: Examining oxyhydrogen gas generation experimentally using wet cell design
Source: PLoS One. 2025 Jun 3;20(6):e0324921. doi: 10.1371/journal.pone.0324921 (PMC12133180; doi:10.1371/journal.pone.0324921)
Supplement: S1 File — S1 Table. Effect of voltage variation on flow rate. S2 Table. Effect of current variation on flow rate. S3 Table. Effect of voltage variation on cell efficiency. S4 Table. Effect of current variation on cell efficiency. S5 Table. Effect of operating time on current. S6 Table. Effect of operating time on HHO flow rate. S7 Table. Effect of cell gap on HHO flow rate. (PDF) [file pone.0324921.s001.pdf]

Effect of voltage variation on flow rate

Test	*Current (A)*	*Voltage (V)*	Actual flow rate Q_{act} (ml/min)	*Theoretical flow rate Q_{th} (ml/min)*
**1**	**7**	**2**	**156**	**285.36**
**2**	**7**	**3**	**215**	**429.69**
**3**	**7**	**4**	**306**	**574.02**
**4**	**7**	**5**	**357**	**718.35**
**5**	**7**	**6**	**410**	**862.68**
**6**	**7**	**7**	**435**	**1007.013**
**7**	**7**	**8**	**456**	**1151.67**
**8**	**7**	**9**	**485**	**1296.07**
**9**	**7**	**10**	**504**	**1440.337**
**10**	**7**	**11**	**528**	**1584.76**

Effect of current variation on flow rate

Test	Voltage (V)	*Current (A)*	Actual flow rate Q_{act} (ml/min)	*Theoretical flow rate Q_{th} (ml/min)*
**1**	***2***	***6***	***160***	***229.55***
**2**	***2***	**10**	**295**	**388.89**
**3**	***2***	**14**	**402**	**548.24**
**4**	***2***	**18**	**490**	**707.585**
**5**	***2***	**22**	**545**	**866.93**
**6**	***2***	**26**	**605**	**1026.275**
**7**	***2***	**30**	**655**	**1185.62**
**8**	***2***	**34**	**696**	**1344.96**
**9**	***2***	**38**	**735**	**1504.31**
**10**	***2***	**42**	**760**	**1663.655**

Effect of voltage variation on cell efficiency

**Test**	***Current (A)***	***Voltage (V)***	**Efficiency %**
**1**	**7**	**2**	***48.02***
**2**	**7**	**3**	**50.3**
**3**	**7**	**4**	**53.89**
**4**	**7**	**5**	**49.72**
**5**	**7**	**6**	**47.56**
**6**	**7**	**7**	**43.154**
**7**	**7**	**8**	**39.58**
**8**	**7**	**9**	**37.4**
**9**	**7**	**10**	**34.9**
**10**	**7**	**11**	**33.33**

Effect of current variation on cell efficiency

**Test**	**Voltage (V)**	***Current (A)***	**Efficiency %**
**1**	***2***	***6***	***69.7***
**2**	***2***	**10**	**76.03**
**3**	***2***	**14**	**73.45**
**4**	***2***	**18**	**69.23**
**5**	***2***	**22**	**62.93**
**6**	***2***	**26**	**58.67**
**7**	***2***	**30**	**55.28**
**8**	***2***	**34**	**51.82**
**9**	***2***	**38**	**47.85**
**10**	***2***	**42**	**45.76**

Effect of operating time on current

Operating time (min)	*NaOH concentration*	*Voltage (V)*	*current (A)*
**cell current at 10 g/L NaOH**			
0	*5*	*6.25*	*15*
10	5	6.25	15.3
20	5	6.25	15.8
30	5	6.25	17
40	5	6.25	17.4
60	5	6.25	17.6
90	5	6.25	18
120	5	6.25	18
current at 10 g/L NaOH			
0	*10*	*6.25*	*20*
10	10	6.25	20.8
20	10	6.25	21.4
30	10	6.25	22
40	10	6.25	22.7
60	10	6.25	23.15
90	10	6.25	23.5
120	10	6.25	23.5
current at 15 g/L NaOH			
0	15	6.25	22
10	15	6.25	23.5
20	15	6.25	24
30	15	6.25	24.6
40	15	6.25	25
60	15	6.25	25.7
90	15	6.25	26
120	15	6.25	26
cell current at 20 g/L NaOH			
0	20	6.25	23.5
10	20	6.25	24.2
20	20	6.25	25
30	20	6.25	25.6
40	20	6.25	26.1
60	20	6.25	26.7
90	20	6.25	27
120	20	6.25	27

Effect of operating time on HHO flow rate

**Operating time (min)**	***NaOH concentration***	***Voltage (V)***	***current (A)***	***flow rate (ml/min)***
**5 g/L NaOH**				
**0**	***5***	***6.25***	***15***	***795***
**10**	**5**	**6.25**	**15.3**	**886**
**20**	**5**	**6.25**	**15.8**	**920**
**30**	**5**	**6.25**	**17**	**945**
**40**	**5**	**6.25**	**17.4**	**960**
**60**	**5**	**6.25**	**17.6**	**968**
**90**	**5**	**6.25**	**18**	**975**
**120**	**5**	**6.25**	**18**	**975**
10 g/L NaOH				
**0**	***10***	***6.25***	***20***	***900***
**10**	**10**	**6.25**	**20.8**	**1035**
**20**	**10**	**6.25**	**21.4**	**1060**
**30**	**10**	**6.25**	**22**	**1095**
**40**	**10**	**6.25**	**22.7**	**1120**
**60**	**10**	**6.25**	**23.15**	**1145**
**90**	**10**	**6.25**	**23.5**	**1160**
**120**	**10**	**6.25**	**23.5**	**1160**
15 g/L NaOH				
**0**	**15**	**6.25**	**22**	**1005**
**10**	**15**	**6.25**	**23.5**	**1166**
**20**	**15**	**6.25**	**24**	**1195**
**30**	**15**	**6.25**	**24.6**	**1227**
**40**	**15**	**6.25**	**25**	**1254**
**60**	**15**	**6.25**	**25.7**	**1300**
**90**	**15**	**6.25**	**26**	**1325**
**120**	**15**	**6.25**	**26**	**1325**
20 g/L NaOH				
**0**	**20**	**6.25**	**23.5**	**1110**
**10**	**20**	**6.25**	**24.2**	**1185**
**20**	**20**	**6.25**	**25**	**1235**
**30**	**20**	**6.25**	**25.6**	**1277**
**40**	**20**	**6.25**	**26.1**	**1315**
**60**	**20**	**6.25**	**26.7**	**1358**
**90**	**20**	**6.25**	**27**	**1375**
**120**	**20**	**6.25**	**27**	**1375**

Effect of cell gap on HHO flow rate

Operating time (min)	*NaOH concentration*	*Voltage (V)*	*current (A)*	*electrolyte temp.*
5 g/L NaOH				
0	*5*	*6.25*	*15*	26
10	5	6.25	15.3	29
20	5	6.25	15.8	33
30	5	6.25	17	33.2
40	5	6.25	17.4	33.9
60	5	6.25	17.6	34.6
90	5	6.25	18	35
120	5	6.25	18	35
10 g/L NaOH				
0	*10*	*6.25*	*20*	28.5
10	10	6.25	20.8	32
20	10	6.25	21.4	37.9
30	10	6.25	22	40.5
40	10	6.25	22.7	41.4
60	10	6.25	23.15	43
90	10	6.25	23.5	44
120	10	6.25	23.5	44

Electrolyte temperature (℃)	Cell gap, mm	HHO gas production rate (ml/min)	
		5% NaOH	10% NaOH
Wet cell at 1 mm cell gap			
30	1	795	900
35	1	886	1035
40	1	920	1060
45	1	945	1095
50	1	960	1120
55	1	968	1145
60	1	985	1160
Wet cell at 3 mm cell gap			
30	3	835	945
35	3	905	1070
40	3	940	1104
45	3	966	1145

**50**	**3**	**988**	**1168**
**55**	**3**	**1021**	**1197**
**60**	**3**	**1040**	**1218**
**Wet cell at 4 mm cell gap**			
**30**	**4**	**845**	**968**
**35**	**4**	**915**	**1115**
**40**	**4**	**966**	**1136**
**45**	**4**	**975**	**1178**
**50**	**4**	**992**	**1195**
**55**	**4**	**1030**	**1235**
**60**	**4**	**1055**	**1266**
**Wet cell at 7 mm cell gap**			
**30**	**7**	**815**	**914**
**35**	**7**	**895**	**945**
**40**	**7**	**920**	**967**
**45**	**7**	**946**	**998**
**50**	**7**	**963**	**1035**
**55**	**7**	**997**	**1087**
**60**	**7**	**1015**	**1125**
